# Supplementary material for: A Food Waste-Derived Organic Liquid Fertiliser for Sustainable Hydroponic Cultivation of Lettuce, Cucumber and Cherry Tomato
Source: Foods. 2023 Feb 7;12(4):719. doi: 10.3390/foods12040719 (PMC9956311; doi:10.3390/foods12040719)
Supplement: Supplementary file 1 [file foods-12-00719-s001.zip › foods-2166206-supplementary.pdf]

*Supplemntary Data*

**Table S1.** Nutrients (N and P) concentrations in lettuce, cucumber and tomato, grown using FoodLift and CLF.

| Plant | Type | Component | Average macro-nutrients concentration on dry weight basis, g/kg | |
|---|---|---|---|---|
| | | | **N (SD)** | **P (SD)** |
| **Lettuce** | **CLF** | Leaves | 258.3 (39.6) | 86 (8.5) |
| | | Roots | 251.3 (29.7) | 88 (5.6) |
| | | Stem | 53 (4) | 11.2 (2.6) |
| | | Stalk | 223.3 (2.1) | 88.2 (1.9) |
| | **FoodLift** | Leaves | 250 (29.5) | 87 (1.6) |
| | | Roots | 261.3 (22.5) | 87 (1.7) |
| | | Stem | 54 (13) | 12.9 (0.2) |
| | | Stalk | 241.7 (22) | 86.7 (5.1) |
| **Cucumber** | **CLF** | Fruit | 27 (7) | 6.9 (5.3) |
| | | Leaves | 35 (6) | 32.7 (3.1) |
| | | Roots | 35 (6.6) | 10 (1) |
| | | Stem | 32 (5.3) | 8.9 (2) |
| | | Stalk | 30.3 (2.5) | 8.3 (0.6) |
| | **FoodLift** | Fruit | 28 (4) | 10 (0.9) |
| | | Leaves | 34 (4) | 30.7 (3.2) |
| | | Roots | 35 (1.5) | 9.8 (0.3) |
| | | Stem | 33 (6.6) | 10 (0.9) |
| | | Stalk | 31 (1) | 6.9 (1) |
| **Tomato** | **CLF** | Fruit | 3 (0.4) | 32.3 (1.5) |
| | | Leaves | 1 (0.5) | 4 (0.5) |
| | | Roots | 18.3 (2.5) | 6.3 (0.6) |
| | | Stem | 5.2 (0.8) | 3.9 (0.3) |
| | | Stalk | 7 (1.1) | 4.7 (1.2) |
| | **FoodLift** | Fruit | 3.1 (0.4) | 31 (1) |
| | | Leaves | 1 (0.4) | 4.8 (0.3) |
| | | Roots | 36 (1) | 5.9 (0.1) |
| | | Stem | 3 (0.7) | 3.9 (0.9) |
| | | Stalk | 9.4 (1.4) | 4.8 (0.7) |

Note: Standard deviation (SD) values are given in parenthesis.

**Table S2.** Cations concentrations in lettuce, cucumber and cherry tomato, grown using FoodLift and CLF.

| Plant | Type | Component | Average cations concentration on dry weight basis, g/kg | | |
|---|---|---|---|---|---|
| | | | Ca (SD) | K (SD) | Mg (SD) |
| Lettuce | CLF | Leaves | 5.2 (1) | 14.3 (1.3) | 1 (0.2) |
| | | Roots | 8.1 (0.8) | 7.5 (0.5) | 36.7 (2.1) |
| | | Stem | 4.3 (0.3) | 4.1 (0.6) | 1.9 (0.3) |
| | | Stalk | 3.3 (0.6) | 3 (0.6) | 0.8 (0.1) |
| | FoodLift | Leaves | 7 (0.2) | 7 (1) | 3.1 (0.7) |
| | | Roots | 5.9 (0.5) | 6.9 (0.7) | 22 (1) |
| | | Stem | 3 (0.2) | 13 (1) | 0.8 (0.1) |
| | | Stalk | 4.3 (0.3) | 20 (1) | 2 (0.2) |
| Cucumber | CLF | Fruit | 1.9 (0.2) | 17.1 (1.4) | 1.9 (0.3) |
| | | Leaves | 18 (1) | 14.4 (2.7) | 7 (0.2) |
| | | Roots | 28.4 (3.5) | 55.1 (3.5) | 7 (1.0) |
| | | Stem | 8 (1) | 16.4 (1.8) | 3 (0.2) |
| | | Stalk | 6.4 (0.7) | 18 (1) | 3 (0.6) |
| | FoodLift | Fruit | 17.8 (1.3) | 20.7 (2.5) | 12 (1) |
| | | Leaves | 2 (0.2) | 17 (1) | 2 (0.2) |
| | | Roots | 3 (0.2) | 30.7 (3.8) | 3 (0.2) |
| | | Stem | 8 (1) | 12 (1) | 18.8 (2.9) |
| | | Stalk | 6.2 (0.9) | 28.7 (5.7) | 3 (0.2) |
| Tomato | CLF | Fruit | 3.1 (0.7) | 17.7 (1.2) | 2 (0.2) |
| | | Leaves | 1 (0.2) | 13 (1) | 1 (0.2) |
| | | Roots | 18 (1) | 20 (1) | 8 (1) |
| | | Stem | 5.4 (0.6) | 2.4 (0.4) | 4.3 (0.3) |
| | | Stalk | 7.2 (0.8) | 37 (1) | 3.4 (0.5) |
| | FoodLift | Fruit | 3.4 (0.5) | 4 (1) | 1.1 (0.1) |
| | | Leaves | 1.1 (0.1) | 21.3 (5) | 14.4 (2.7) |
| | | Roots | 35.7 (1.5) | 29.5 (1.7) | 2 (0.2) |
| | | Stem | 3.1 (0.4) | 54.2 (2.4) | 14.4 (2.7) |
| | | Stalk | 9 (0.2) | 12 (1) | 1.9 (0.2) |

Note: Standard deviation (SD) values are given in parenthesis.
